# Physical Activity Promotion Programmes in Childhood Cancer Patients and Their Impact on Fatigue and Pain: A Systematic Review

**DOI:** 10.3390/children8121119

**Published:** 2021-12-02

**Authors:** Catherine Malysse, Rita Pilar Romero-Galisteo, Jose Antonio Merchán-Baeza, J. Ignacio Durán-Millán, Manuel González-Sánchez, Alejandro Galan-Mercant

**Affiliations:** 1 Department of Physiotherapy, Faculty of Health Sciences, University of Málaga, Avda. Cervantes, 2, 29071 Málaga, Spain; cathymalysse8@gmail.com (C.M.); jidm@uma.es (J.I.D.-M.); mgsa23@uma.es (M.G.-S.); 2 Instituto de Investigación Biomédica de Málaga, IBIMA, 29010 Málaga, Spain; 3 Research Group on Methodology, Methods, Models and Outcomes of Health and Social Sciences (M_3_O), Centre for Health and Social Care Research (CESS), Faculty of Health Science and Welfare, University of Vic-Central University of Catalonia (UVIC-UCC), C. Sagrada Família, 7, 08500 Vic, Spain; josan.merchan@uvic.cat; 4 MOVE-IT Research Group, Department of Physical Education, Faculty of Nursing and Physiotherapy, University of Cádiz, 11003 Cádiz, Spain; alejandro.galan@gm.uca.es; 5 Biomedical Research and Innovation Institute of Cádiz (INiBICA) Research Unit, Puerta del Mar University Hospital (Cádiz), University of Cádiz, 11002 Cádiz, Spain

**Keywords:** child, cancer, fatigue, pain, health promotion, physical activity, coaching

## Abstract

Cancer is one of the main causes of death in children, however, the techniques and interventions applied allow the cure of 80% of diagnosed cases. The aim of this review was to determine the benefits of a health and physical activity promotion programme to reduce pain and fatigue symptoms in children and adolescents with cancer. The databases PubMed, Embase, Scopus, Cochrane, Web of Science and PEDro were searched between December 2020 and January 2021 to elaborate this review, using the keywords child, cancer, exercise, fatigue and pain. The review was preregistered in PROSPERO (ID CRD42021262183). Six studies, out of 937 identified at baseline, were finally included in the review: four randomised controlled trials and two quasi-experimental studies. The total sample size of all the included studies was of 474 participants with very different types of cancer and evolution, and outcome variables were pain, fatigue, physical activity level, self-efficacy and quality of life. A health and physical activity promotion programme seems to improve fatigue in paediatric cancer patients and survivors, but no significant results were found related to pain.

## 1. Introduction

Childhood cancer refers to any cancer type in children and adolescents aged 0 to 18 [1]. Even though it is a disease with a low incidence in certain countries such as Spain, with approximately 1000 new cases every year, childhood cancer is one of the main causes of infant death worldwide [1,2,3,4]. Fortunately, thanks to new diagnostic technologies, treatments and techniques, approximately 80% of all children diagnosed with cancer are cured in five or more years following the diagnosis [1,5,6].

The most frequent types of cancer in the paediatric population include leukaemia (which accounts for approximately 30% of all cases), lymphomas and tumours of the central nervous system, the prevalence of which varies according to the age of the patient. For example, in the USA, acute lymphoblastic leukaemia is more common in children (26%), while Hodgkin’s lymphoma is more prevalent in adolescents (15%) [7,8].

It is undeniable that greater cancer survival rate is a positive fact, but it also means that more people suffer severe adverse effects due to the treatment, which are even more severe in young patients [9,10]. However, these adverse effects do not occur exclusively during the disease, but also in cancer survivors as cardiotoxicity is one of the main morbidity causes due to chemotherapy treatments with anthracyclines and radiation [11,12]. Furthermore, the tendency to a sedentary lifestyle increases other risk factors such as risk of overweight or obesity, arterial hypertension, muscular atrophy, etc., which can drastically reduce the patient’s quality of life (QOL) [10]. Apart from other reasons, this is why the risk of early mortality is eight times higher in these patients compared to the general population [11,13].

A qualitative synthesis of different systematic reviews by Stout et al. [14] grants physical activity (PA) a strong validity to be included in treatment and care programmes since it can help decrease these comorbidities and death risks [15]. Nevertheless, there are multiple factors that can make exercise difficult in childhood population, such as long hospitalisation periods or fatigue [16], which imply a larger tendency to abandon PA. Furthermore, parents’ fear can also negatively influence the treatment [17]. This is why childhood cancer requires a global approach, such as the ICF-CY (International Classification of Functioning, Disability and Health for Children and Youth) [18] that advocates important changes in the physical, social and psychological development of these young children, as well as environmental changes depending on their age.

It is known that fatigue is one of the factors that greatly influences the abandoning of physical activity, as mentioned above. Hence, the importance of developing motivating and fun exercise programmes according to the age of the paediatric patient [19]. On the other hand, early interventions aimed at mitigating pain have the potential to prevent the onset of chronic pain and psychiatric comorbidities in adulthood [20], as this population suffers some severe adverse effects even up to 30 years after diagnosis.

Physical activity promotion programmes aim to build self-efficacy and self-determination in the individual to lead a healthy lifestyle. These programmes can be carried out directly by professionals or indirectly by educating the children’s family members or caregivers. This promotes not only the self-efficacy and adherence to treatment of the child, but also of the parents, which implies increased supervision and therefore probably improved outcomes [21].

The purpose of this study is to determine the effectiveness of a PA and health promotion programme in order to improve pain and fatigue symptoms in child cancer patients, as we are not aware of any review dealing with this issue to this date.

## 2. Materials and Methods

This systematic review was conducted through a literature search between December 2020 and January 2021.

### 2.1. Search Strategy

The bibliographic search was carried out using the following databases: PubMed, Embase, Scopus, PEDro, Web of Science and Cochrane. The keywords used in every database were “child*”, “cancer”, “exercise”, “fatigue” and “pain”, using “AND” and “OR” combinedly as Boolean operators. Two different searches were carried out with these keywords: a first search without any filters in all databases except Cochrane, and a second one using “clinical trials” as a filter.

### 2.2. Inclusion and Exclusion Criteria

**Figure 1:**
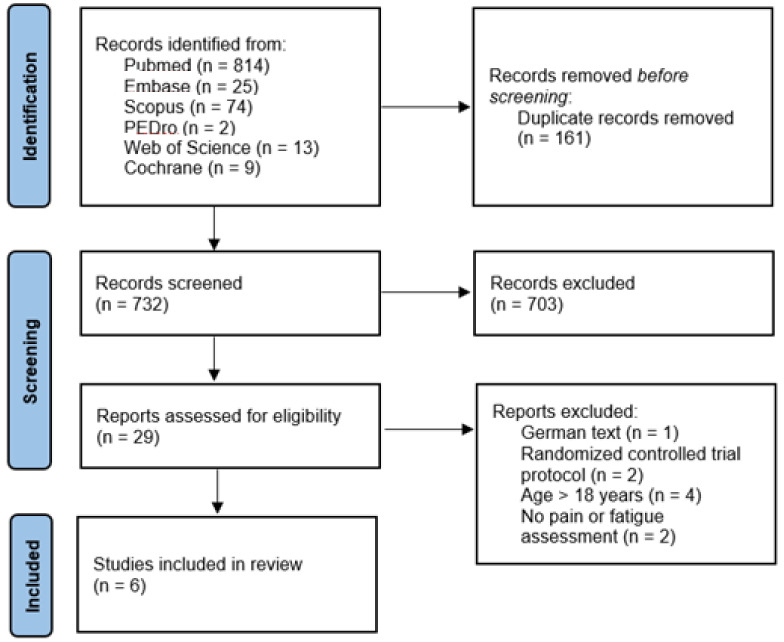
PRISMA flow diagram.

Inclusion criteria were: (a) randomized controlled trials or quasi-experimental studies; (b) published in English, Spanish, French or Dutch; (c) with pain or fatigue as outcome variables; (d) in child cancer patients or survivors; (e) aged 0–18; (f) who were included in a health and PA promotion programme. Only articles that did not report results, such as study protocols, were excluded. All search results were included regardless of their publication date, and the PRISMA methodology was followed to reflect them (Figure 1) [22].

### 2.3. Procedure

Two blinded reviewers (C.M. and R.R.) carried out the bibliographic searches independently of one another using the previously mentioned databases. All duplicates were eliminated, and inclusion and exclusion criteria were checked by reading the abstracts of each of the remaining articles. After the first screening by two of the reviewers (R.R. and M.G.), a third reviewer (C.M.) proceeded to resolve the discrepancies and doubts raised during the selection of the different articles, as well as to read each of the remaining articles independently to determine the eligibility criteria and discard all articles not related to the purpose of this review. Once the different articles were selected, an evaluation of their internal validity was carried out using the PEDro scale.

The study protocol of the present review has been registered in the PROSPERO database of the University of York.

### 2.4. Methodological Quality Assessment

The PEDro scale was used to assess the methodological quality of the RCTs included in this review [23]. The PEDro score has demonstrated ‘fair’ to ‘excellent’ inter-rater reliability (Intraclass Correlation Coefficient 0.53–0.91) for RCTs of physiotherapy interventions [24]. While these authors report that total PEDro scores of 0–3 are considered ‘poor’, 4–5 ‘fair’, 6–8 ‘good’, and 9–10 ‘excellent’, it is important to note that these classifications have not been validated. Furthermore, for trials evaluating complex interventions (e.g., exercise) a total PEDro score of 8/10 is optimal.

## 3. Results

A total number of 937 articles were identified. After a first screening, 44 articles were selected for full-text reading, and after eliminating a few studies that did not meet the inclusion criteria, 29 articles were evaluated. Finally, six documents were selected for this qualitative synthesis (Figure 1).

Table 1 describes the studies analysed in this systematic review. The total participants sample size of this review is 474 study subjects. Nevertheless, the distribution of these sample sizes is not homogenous as almost half of the total sample size (222 patients) relates to the study by Li et al. [25], while the remaining articles have sample sizes of less than 100 participants. There is a wide variety of cancer types between the selected articles: while some discussed a specific type of cancer, most of the studies did not set a concrete cancer type as an inclusion criterion. Furthermore, in the case of the clinical trials by Mendoza et al. [26] and Li et al. [25], survivors of any type of childhood cancer were selected. In the penultimate column of Table 1, the types of intervention undergone by the experimental and control groups of each of the different studies are stated.

The PEDro scale was used to evaluate the methodological quality of the different studies included in this review, although only four of them [25,26,27,28] were assessed on the basis of this scale as the two remaining articles [29,30] were quasi-experimental studies. In the last column of Table 1, the quantitative values of the methodological quality of these four RCTs [25,26,27,28] are stated on the basis of this scale, with a score of 0–10 according to its criteria. The articles written by Lam et al. [28] and Li et al. [25] had the highest quality with a score of 7/10, while the study by Mendoza et al. [26] obtained the lowest score with 5/10. None of these four articles scored on the blinding of patients or therapists, but due to the nature of these interventions, this blinding is impossible. Therefore, the maximum achievable score would be 8/10.

Taking this into account, it can be concluded that the studies by Lam et al. [28] and Li et al. [25] have a high methodological quality and the remaining ones a slightly inferior one, although none of them obtained a score lower than 5/10. Regarding the items of this scale, it is noteworthy that three out of these four articles follow an intention-to-treat analysis [25,26,28], which means that no study subjects were lost when interpreting the results. This is also reflected in the values of Table 2.

Table 2 shows that the duration of the interventions in the different studies varies between 10 weeks (Mendoza et al. [26]) and 135 weeks (Cox et al. [27]) since the initial assessment, while the remaining studies had a duration between 6 and 12 months [25,28,29,30]. The results of the analysed outcome variables of every article related to the aim of this study, specifically fatigue [25,28,29,30], pain [26,27], PA level [25,26,27,28,29,30], QOL [25,26,27,28,30] and self-efficacy level to undertake PA [25,28], are also stated.

## 4. Discussion

This review was designed to test the effectiveness of a health and physical activity education programme to improve fatigue and pain symptoms in child cancer patients and/or survivors. The outcomes suggest that this intervention improves fatigue, nevertheless, it does not lead to changes in pain symptoms. This analysis is, to our knowledge, one of the few that assesses the effectiveness of this type of intervention on the outcome variables previously described.

### 4.1. Effects on Pain Symptoms

While assessing the effects of this intervention on pain symptoms, an important barrier has been identified, as only two of the six studies included in this review assessed pain as an outcome variable [26,27], and none of them as a primary outcome, but rather as an item of a QOL questionnaire: “Child Health Questionnaire” (Cox et al. [27]) and “PedsQL Cancer Module Scales” (Mendoza et al. [26]). In addition to using different assessment scales, these articles also evaluate two completely different study populations: while Cox et al. [27] focus on patients with acute lymphoblastic leukaemia undergoing cancer treatment, Mendoza et al. [26] assess 14–18-year-old adolescent cancer survivors. Therefore, it is more likely that participants in the second study did not suffer from cancer-associated pain in their daily lives, which leaves us with only one clinical trial that assesses pain in children undergoing cancer treatment. In this study, only slight improvements were found in the PA levels of both the control and experimental groups, but none were found in pain levels. However, one thing to note from the study by Cox et al. [27] is that the parents’ estimation of the pain of the children who belonged to the experimental group at the 135-week assessment showed a minimal difference to the symptoms reported by the patients, while this did not occur in the first assessments. This data thus supports McWilliams family-centred intervention model [31], as the intervention of this study resulted in the parents’ better knowledge of their children’s condition.

### 4.2. Effects on Fatigue

Three out of the four articles included in this review that assessed fatigue reported improvements in the fatigue levels of patients who received a PA education programme [25,28,30], while the study by Hooke et al. [29] did not find these same improvements despite using the same measuring instrument (FS-C) as Lam et al. [28] and Li et al. [25]. When comparing the descriptive data and outcomes of the studies by Hooke et al. [29], Lam et al. [28] and Li et al. [25], several reasons can be identified why there might not have been changes in the fatigue levels of the participants in the first study. Firstly, the authors of this article state that the health workers in charge of developing the programme hardly implemented the coaching treatment due to patients’ adverse reactions to the cancer treatment such as neuropathies and muscle weakness, and consequently, they had to be referred to physiotherapy [29]. Furthermore, if we compare the sample sizes of these three studies, the article by Hooke et al. [29] has the smallest one and also has a dropout rate larger than 50% due to various reasons such as the completion of cancer treatment between the 4th and 6th assessment months, thus leaving patients with worse outcomes in the study.

Out of the three articles that did find significant differences in patients’ fatigue levels [25,28,30], two of them did not only use the same tool to measure this outcome variable (Chinese version of the FS-C), but also the same measuring instruments for all the other variables under analysis [25,28]. If we analyse the outcomes of both studies, we can see that the fatigue levels decreased significantly earlier in time in the clinical trial by Lam et al. [28] than in the trial by Li et al. [25]. This could be due to two important factors: first of all, Lam et al. [28] evaluated patients undergoing cancer treatment, which implies a greater scope of improvement than in cancer survivors. Furthermore, the fatigue levels of the control group, who did not receive a coaching intervention, generally worsened due to the chemotherapy treatment, which increases the difference between fatigue outcomes in both groups. Secondly, the intervention by Lam et al. [28] was much more coaching-oriented and had a higher frequency of sessions, and therefore of supervision, than in the Li et al. [25] study.

The quasi-experimental study by Ovans et al. [30] also found statistically significant differences in fatigue levels using the PedsQL Multidimensional Fatigue Scale. However, no improvement was found in the PA levels in steps/day recorded by the FitBit Flex^®^ worn by the study participants during the intervention period. This may be due to two factors: firstly, the ongoing treatment against the brain tumour and secondly, a low frequency and duration of the coaching sessions. Nevertheless, patients reported improvements, though not statistically significant, in their subjective perception of PA levels, and a statistically significant improvement in total, general and sleep/rest fatigue was detected after the first assessment. Therefore, even though these patients did not objectively improve their PA levels, they had a subjective perception of improvement and furthermore, their fatigue levels also decreased significantly. This raises suspicions of bias, as the patients knew the aim of the study since the measuring instruments of both variables were subjective, or of a possible placebo effect. It is therefore interesting to use, if possible, both a subjective and an objective measuring instrument to assess the presence of this factor.

The measuring instruments used in the different studies were diverse: for fatigue assessment, the most commonly used questionnaire was the FS-C [25,28,29], while the research by Ovans et al. [30] used the PedsQL Multidimensional Fatigue Scale, which divides the fatigue assessment into three components: general, sleep/rest and cognitive fatigue. However, for pain assessment, the studies did not use the same measuring instruments: while Cox et al. [27] used the Child Health Questionnaire, which apart from pain also determines patients’ QOL and parents’ estimation of their children’s QOL, Mendoza et al. [26] used the PedsQL Cancer Module Scores. Throughout all studies, the measurement of the PA levels was carried out either subjectively, using questionnaires such as the modified Leisure Score Index of the GLTEQ [29,30] or the CUHK-PARCY [25,28], or objectively through the use of accelerometers (Actigraph GT3X [26,29], SenseWear Pro III [27] and FitBit Flex^®^ [30]); and the PA-SE scale was used to measure patients’ self-efficacy levels to perform PA [25,28]. Lastly, the main measuring instrument used to assess study participants’ general QOL was the PedsQL scale [25,26,28,30].

Therefore, none of the measurement instruments simultaneously assessed the effect of the intervention on fatigue and pain outcomes. A study with a similar goal by Van Dijk-Lokkart et al. [32] did assess both fatigue and pain using some of the previously mentioned assessment tools, but due to the lack of a PA promotion programme, this study has not been included in the present review.

Another limitation of this review is the lack of comparison of the effectiveness of a combined PA and health promotion treatment with a physical activity-only intervention, which means that the extent to which the PA promotion and motivation programme is more beneficial to patients than a normal exercise programme is not known. Furthermore, the health promotion programmes designed by the different articles included in this review differ: while three of the studies used coaching as a way to promote PA [28,29,30], the three remaining ones only used the term “health promotion” as part of their intervention [25,26,27], without providing a specific definition of the term. In addition, among the articles that used coaching as an intervention, no common conceptual framework was described, nor were the steps to be taken in order to achieve the aim of this intervention defined. However, health promotion programmes are not the only factor with respect to which there is a discrepancy between the articles included in this review, as there also exists a wide range of pathological conditions within child cancer: while two of the articles studied the effects of the promotion programme on childhood cancer survivors [25,26], the other four evaluated patients under treatment [27,28,29,30], either for a specific type of cancer, such as brain tumours [30] or acute lymphoblastic leukaemia [27], or for different types of cancer in general [28,29]. This wide pathological range implies a wide variety of treatments and therefore a lack of consideration of possible adverse reactions to treatment of a group of patients with a specific type of cancer, which may influence the study outcomes. Furthermore, considering the age difference between study participants and their growth stages, hormonal changes and personal preferences, there are many factors beyond the authors’ control that could influence the results.

Nevertheless, the strengths of this review are also worth mentioning, as the included articles are recent since the oldest dates from 2017 [26] and the most recent from 2019 [29]. In addition, this article is, to our knowledge, one of the few that evaluates the effectiveness of a PA promotion programme on pain and fatigue in paediatric oncology, and the methodologic quality of the RCTs included is quite good considering that the highest achievable score on the PEDro scale is 8/10.

## 5. Conclusions

In conclusion, a health and PA promotion programme such as coaching can reduce fatigue levels and, consequently, increase childhood cancer patients’ and survivors’ QOL. However, no results have been found to indicate that this programme leads to an improvement in pain. These results have to be carefully interpreted due to the limited number of clinical trials included in this review and, furthermore, they cannot be generalised. This is why many more clinical trials are needed to evaluate the different types of childhood cancer more specifically, in order to offer these young patients strategies that address their problems in a personalised way to optimise the therapeutic process.

## Figures and Tables

**Table 1:** Characteristics and methodological quality of the studies analysed according to the PEDro scale.

Author and Year	Sample Size	Study Design	Cancer Type	Age	Intervention	PEDro Scale
Cox et al. [27] (2018)	n = 77: - E.G.: 36 - C.G.: 41	Randomized controlled trial (RCT)	Acute lymphoblastic leukaemia	4–18 years	E.G.: - 30 min personalised PA routine 5 days/week aimed at strengthening, improvement of ROM, of gross motor function and endurance, together with a PA education programme for patients and their family members through 37 home visits or phone calls by physical therapists. - 43 home visits or phone calls by nurses with strategies aimed at supporting motivation and long-term behavioural changes for patients and their family members. C.G.: - Attention-control home visits or phone calls by physical therapists at the same time interval as the E.G., with recommendations of passive ankle stretching to slow down the limitation of ankle ROM due to the pharmacological treatment with vincristine.	6/10
Hooke et al. [29] (2019)	n = 30	Quasi-experimental study with a nonrandomized comparative group design	- Acute lymphoblastic leukaemia (n = 7). - Solid tumours (n = 12). - Lymphoma (n = 11).	6–18 years	“Kids Are Moving” programme starting the second month of cancer treatment. This programme is based on typical PA for children, such as playing hide and seek or riding a bike, and was implemented in combination with PA coaching in five steps: determining the patients’ current PA level, assessing possible health barriers to PA, determining the stage of change the patients and their caretakers are in, writing the personalised PA prescription with recommendations on frequency and intensity, and providing information on resources.	N/A
Lam et al. [28] (2018)	n = 70 - E.G.: 37 - C.G.: 33	Randomized controlled trial (RCT)	Not specified	9–18 years	E.G.: - An integrated experiential training programme guided in 28 1-h home visits by student nurses, combined with a coaching programme aimed at increasing the patients’ PA and self-efficacy levels through the setting of new challenging goals and a series of difficult physical activities. PA focused mainly on aerobic, resistance, stretching and relaxation exercise of mild-moderate intensity. C.G.: - Home visits at similar time intervals as the E.G., with placebo interventions which do not have any specific effects on the outcome measures: chess and card games, providing health advice on the importance of a healthy diet, etc. Both groups: - 15 min health education talk during hospitalisation. - 30 min individual English tutorials during the home visits to promote treatment adherence.	7/10
Li et al. [25] (2018)	n = 222 - E.G.: 117 - C.G.: 105	Randomized controlled trial (RCT)	Child cancer survivors (type not specified)	9–16 years	E.G.: - 4 sessions of an adventure-based training programme (2 weeks–2 months–4 months–6 months after randomisation) with a previous 45 min briefing session including health education components. Each exercise in the training programme was designed with a different objective in mind: improving muscle strength and vital capacity, promoting self-esteem, empowering the patient, … C.G.: - Placebo intervention at the same time intervals as the E.G., without any specific effects on the outcome measures: board games, movies, museum visits, …	7/10
Mendoza et al. [26] (2017)	n = 60 - E.G.: 30 - C.G.: 30	Pilot randomized controlled trial (RCT)	Child cancer survivors (type not specified)	14–18 years	E.G.: - A 10-week PA promotion programme through the use of a FitBit Flex^®^. The research staff set new daily step goals every week based on the mean daily step number of the previous week, and gradually increased their step goal in the following weeks until reaching or maintaining the general step recommendations for adolescents. - Participation in a Facebook group created specifically for this RCT, composed by study participants and moderated by research staff members. This group was dedicated to motivating and reminding patients of their PA target, as well as sharing their personal experiences. Participation in both interventions was voluntary in order to obtain real-world feasibility. C.G.: - Advice on PA for childhood cancer survivors and its importance to their health. The C.G. did not receive an active intervention.	5/10
Ovans et al. [30] (2018)	n = 15	Quasi-experimental study	Brain tumours	7–18 years	A 12-week intervention combining: - The use of a FitBit Flex^®^ with a personalised step goal based on the average steps of the previous days and the daily step recommendations for healthy children. - Coaching by physical therapists every 2–3 weeks (a total number of 5 coaching sessions): count of the average number of steps of the previous days, patients’ progress, identifying strategies to increase patients’ PA level in the coming weeks, possible barriers to PA and how to overcome them. After each coaching session, a new step goal was suggested and programmed into the FitBit Flex^®^.	N/A

Abbreviations: C.G.: control group; E.G.: experimental group; N/A: not applicable; n: sample size; P.A.: physical activity; ROM: range of movement.

**Table 2:** Outcome measures of the analysed studies.

Author and Year	Time of Assessment	Outcome Variables	Assessment Tools	Outcomes: Mean (SD)						Conclusions
				Groups	T0		T1	T2	T3	
Cox et al. [27] (2018)	T0: initial assessment (n = 107). T1: 8 weeks (n = 97). T2: 15 weeks (n = 92). T3:135 weeks (n = 77).	Health-related quality of life (includes pain): patients and parents.	Patients: Child Health Questionnaire ^a^ (bodily pain)	E.G.	59.58 (28.36)		55.58 (25.19)	60.73 (25.53)	71.58 (23.31)	Although there were improvements in outcomes over time, there were no statistically significant differences between the two groups
				C.G.	56.38 (26.82)		65.23 (26.37)	66.67 (25.05)	76.34 (22.78)	
			Parents: Child Health Questionnaire (bodily pain)	E.G.	46.79 (24.24)		46.6 (19.14)	58.37 (19.02)	71.08 (23.78)	
				C.G.	49.81 (23)		50.42 (16.01)	60.21 (22.55)	72.38 (25.26)	
		PA patterns (hours/day)	SenseWear Pro III accelerometer	E.G.	-		8.45 (9.94)	5.81 (5.91)	11.86 (10.06)	
				C.G.	-		9.32 (12.26)	7.84 (10.17)	12.84 (12.80)	
Hooke et al. [29] (2019)	T1: 2 months (n = 28). T2: 4 months ^b^ (n = 21). T3: 6 months (n = 12).	PA level	Self-report: leisure score index of the GLTEQ	E.G.			54.9 (10.8)	60.4 (7.8)	51.1 (10.2)	No statistically significant differences have been found in the outcomes between both groups of over time.
				Historical C.G ^b^.			-	49.3 (63.1)	-	
			Actigraphy ^c^: Actigraph GT3X accelerometer.	E.G.	Steps/day		4000	3500	4300	
					PA minutes/day		95	70	70	
					% sedentarism/day		80	85	85	
		Fatigue^d^	FS-C^b^ and FS-A ^b^	E.G.	Combined group		53.9 (2.0)	51.0 (2.0)	48.7 (2.6)	
					ALL		59.6 (5.2)	54.6 (4.3)	-	
					Lymphoma		54.3 (2.2)	47.6 (5.2)	-	
					Solid tumours		50.8 (3.8)	55.0 (2.9)	58.7 (2.1)	
				Historical C.G^b^.			-	51.1 (9.1)	-	
Lam et al. [28] (2018)	T0: initial assessment (n = 70). T1: 6 months (n = 70). T2: 9 months (n = 70).	Fatigue	Chinese version of the FS-C	E.G.	49.2 (7.5)		48.2 (7.2)	47.6 (7.5)	-	Statistically significant improvement in all outcome variables of the E.G. from T0 to T2.
				C.G.	49.7 (6.9)		53.7 (7.0)	54.7 (6.7)	-	
		PA level	CUHK-PARCY questionnaire	E.G.	2 (1.2)		-	4 (2.0)	-	
				C.G.	2 (1.3)		-	1.9 (1.3)	-	
		PA self-efficacy	PA-SE scale	E.G.	7.8 (2.3)		8.4 (1.8)	8.6 (2.0)	-	
				C.G.	7.7 (2.7)		6.4 (2.0)	6.3 (2.2)	-	
		QOL	Chinese version of the PedsQL questionnaire	E.G.	63.0 (7.1)		64.0 (6.0)	64.7 (6.0)	-	
				C.G.	62.3 (9.2)		60.4 (9.0)	58.0 (8.5)	-	
Li et al. [25] (2018)	T0: initial assessment (n = 222). T1: 6 months (n = 222). T2: 12 months (n = 222).	Fatigue	Chinese version of the FS-C	E.G.	29.4 (4.2)		26.6 (4.9)	22.3 (4.2)	-	Statistically significant improvement in all outcome variables of the E.G. from T0 to T2.
				C.G.	29.2 (4.1)		28.5 (4.2)	28.9 (4.9)	-	
		PA level	CUKH-PARCY questionnaire	E.G.	3.0 (1.2)		5.2 (1.6)	6.0 (1.8)	-	
				C.G:	3.2 (1.1)		3.3 (1.2)	3.5 (1.6)	-	
		PA self-efficacy	PA-SE scale	E.G.	9.1 (3.4)		10.5 (3.0)	11.9 (3.0)	-	
				C.G.	9.0 (3.1)		9.1 (3.0)	9.0 (3.1)	-	
		QOL	Chinese version of the PedsQL scale	E.G.	68.6 (11.6)		70.3 (11.9)	79.8 (13.2)	-	
				C.G.	68.4 (11.5)		68.4 (12.0)	70.1 (12.8)	-	
Mendoza et al. [26] (2017)	T0: initial assessment (n = 59). T1: 8–10 weeks (n = 59).	PA level	Actigraph GT3X+ ^e^ accelerometer	PA minutes/day	E.G	-	+4.4	-	-	No significant differences were found for any of the outcome variables in either of the two study groups.
					C.G	-	+5	-	-	
				Sedentarism	E.G	-	−4.5	-	-	
					C.G	-	+1	-	-	
		Health-related QOL (includes pain)	PedsQL 4.0 Generic Core ^f^ scale	E.G.	79.7		79.1	-	-	
				C.G.	77.5		79.4	-	-	
			PedsQL Cancer Module Scores ^f^: bodily pain	E.G.	73.2		69.6	-	-	
				C.G.	73.5		70.8	-	-	
Ovans et al. [30] (2018)	T0: initial assessment (n = 15). T1: 12 weeks (n = 15). T2: 24 weeks (n = 11).	PA level	Objective: FitBit Flex^®^ (steps/day)	E.G.	8956 (4589)		8944 (3022)	10,141 (3260)	-	Statistically significant improvements in total, general and sleep/rest-related fatigue between T0 and T1, as well as in total and general fatigue between T0 and T2 of the 11 participants left.
			Subjective: leisure score index of the GLTEQ ^f^	E.G.	45		52	73	-	
		QOL	PedsQL Generic Core Scale ^f^	E.G.	84.38		85.50	91.75	-	
		Fatigue	PedsQL Multidimensional Fatigue Scale ^f^	E.G.	Tot.F.	72.22	83.33	-	-	
					Gen.F.	70.83	83.33	87.50		
					S-R. F.	62.50	75.00	79.17		
					Cog.F.	79.17	83.33	87.50		

Abbreviations: ALL: acute lymphoblastic leukaemia; C.G.: control group; Cog.F.: cognitive fatigue; CUHK-PARCY: Chinese University of Hong-Kong Physical Activity Rating for Children and Youth; E.G.: experimental group; FS-A: Fatigue Scale—Adolescent; FS-C-: Fatigue Scale—Child; Gen.F.: general fatigue; GLTEQ: Godin-Leisure-Time Exercise Questionnaire; PA: physical activity; PA-SE: Physical Activity Self-Efficacy Scale; PedsQL: Paediatric Quality of Life Inventory; n: sample size; QOL: quality of life; SD: standard deviation; S-R. F.: sleep/rest fatigue; T0: initial assessment; T1: first assessment; T2: second assessment; T3: third assessment; Tot.F.: total fatigue. Additional information: ^a^ This questionnaire was not completed by children under 5 years of age. ^b^ These variables were evaluated in a historical control group (n = 27) from the same centre after receiving the cancer intervention (4 months) and before receiving the “Kids Are Moving” programme. ^c^ These outcomes are not presented in a numerical form, but in graphs. ^d^ Fatigue outcomes are presented for both the combined group and for each of the different cancer types. ^e^ These results were analysed by measuring the difference between T0 and T1 of the mean minutes of moderate-vigorous PA and sedentary activity in both groups. ^f^ These results are presented without standard deviation.

## Data Availability

Data is contained within the article.
